# Transgene expression of Stanniocalcin-1 provides sustained intraocular pressure reduction by increasing outflow facility

**DOI:** 10.1371/journal.pone.0269261

**Published:** 2022-05-31

**Authors:** Gavin W. Roddy, Uttio Roy Chowdhury, Kjersten J. Anderson, Tommy A. Rinkoski, Cheryl R. Hann, Vince A. Chiodo, W. Clay Smith, Michael P. Fautsch

**Affiliations:** 1 Department of Ophthalmology, Mayo Clinic, Rochester, Minnesota, United States of America; 2 Department of Ophthalmology, University of Florida, Gainesville, Florida, United States of America; Duke University, UNITED STATES

## Abstract

Glaucoma is the leading cause of irreversible blindness worldwide. Therapies for glaucoma are directed toward reducing intraocular pressure (IOP), the leading risk factor and only reliable therapeutic target via topical medications or with procedural intervention including laser or surgery. Though topical therapeutics are typically first line, less than 50% of patients take drops as prescribed. Sustained release technologies that decrease IOP for extended periods of time are being examined for clinical use. We recently identified Stanniocalcin-1, a naturally occurring hormone, as an IOP-lowering agent. Here, we show that a single injection into the anterior chamber of mice with an adeno-associated viral vector containing the transgene of stanniocalcin-1 results in diffuse and sustained expression of the protein and produces IOP reduction for up to 6 months. As the treatment effect begins to wane, IOP-lowering can be rescued with a repeat injection. Aqueous humor dynamic studies revealed an increase in outflow facility as the mechanism of action. This first-in-class therapeutic approach has the potential to improve care and reduce the rates of vision loss in the 80 million people worldwide currently affected by glaucoma.

## Introduction

Glaucomatous optic neuropathy (GON), also known as glaucoma, remains the world’s leading cause of irreversible blindness and refers to a specific pattern of progressive retinal ganglion cell loss that is pressure-sensitive in nature [1]. Affecting over 80 million people worldwide [2], GON is broadly divided into open-angle and closed-angle glaucoma each with primary and secondary causes. For all of the glaucomas, the only reliable therapeutic target is the reduction of intraocular pressure (IOP), the most prevalent risk factor for GON. Ocular hypertension, or elevated IOP in the absence of GON may also be treated with IOP lowering medications.

IOP is a function of ocular aqueous humor production and drainage. Aqueous humor drainage occurs via two major pathways [3]. The conventional, or trabecular pathway is the predominant pathway that drains the majority of aqueous humor through the trabecular meshwork into a venous plexus in the episcleral venous system which has a basal pressure known as episcleral venous pressure (EVP). The second major pathway is known as the uveoscleral outflow pathway and drains aqueous humor primarily through the ciliary muscle. Treatment strategies for IOP reduction include pharmacologic or laser therapies that try to reduce the production or increase the drainage of aqueous humor. If these modalities are not sufficient, surgical intervention may be required to create a new drainage channel. Use of topical eye drops that contain ocular hypotensive properties are often the initial treatment due to risks associated with procedural intervention [4].

Of the eyedrop therapies, prostaglandin F2α (PGF2α) analogues such as latanoprost or bimatoprost are frequently offered as first line therapy [5]. Though generally well-tolerated, the use of PGF2α analogues are limited in some patients due to their side effect profile that includes ocular surface irritation and intraocular inflammation [6–9]. Furthermore, up to 20% of patients are either minimally responsive or non-responsive to PGF2α analogues, likely due to variants of the cellular receptor for PGF2α analogues, the prostaglandin F (FP) receptor [10–15]. For patients unable to use PGF2α analogues or when single drop therapy is not sufficient for IOP reduction, multiple medication classes, some with up to four times daily dosing regimens, may be prescribed to lower IOP. Unfortunately, less than half of glaucoma patients use therapeutic eye drops as prescribed, mainly due to poor compliance resulting from this complicated dosing schedule, resulting in increased disease progression [16–20]. Furthermore, ocular surface disease may occur in over 50% of glaucoma patients on eye drop glaucoma medications [21, 22] and the condition is more severe with increasing numbers of medications used, number of instillations per day, and use of preservatives in topical medications [23]. Finally, inherent in topical delivery approaches are a pulsatile effect of waxing and waning drug concentration that results in fluctuation of IOP [24]. These fluctuations may be compounded by inconsistency in dose timing and missed doses of medications. Though the absolute value of IOP is a risk factor for progression of GON, fluctuation in IOP levels may be as significant or even more significant in the progression of GON [25].

In order to combat these issues, sustained IOP reduction is an increasingly sought-after therapeutic goal in order to minimize patient non-compliance, ocular surface side effects, and IOP fluctuation. A variety of approaches to deliver available medications have been employed, including contact lens delivery and implants to the conjunctival fornix, lacrimal puncta, periocular space, or anterior chamber (see recent review by Kompella et al.) [24]. However, available approaches require the use of a scaffold to elute drug which may lead to patient discomfort when used extraocularly or complications including corneal endothelial cell loss when used intraocularly. Transgene expression of an IOP-lowering protein would allow for sustained expression of the IOP-lowering compound without use of a scaffold. We recently identified Stanniocalcin-1 (STC-1) as one such candidate molecule.

STC-1, a 50-kDa disulfide-linked dimer with 11 paired cysteine residues, has been shown to function in mineral metabolism and contains anti-inflammatory [26], anti-oxidative stress [27], and neuroprotective [28] properties. STC-1 functions as a stress-response protein being produced at relatively low levels during periods of physiologic homeostasis, but upregulated in times of cellular stress [29] including inflammation [30], oxidation [31], and hypoxia [32–34]. This observation is highlighted by STC-1 knockout mice demonstrating a normal phenotype [35] but develop worse disease compared to controls when subjected to pathologic stress [29, 36, 37]. Our laboratory became interested in STC-1 as a potential IOP-lowering molecule while studying the response of cells involved in aqueous humor drainage following treatment with PGF2α analogue latanoprost free acid (LFA) [38]. STC-1 was one of the most consistently upregulated molecules following LFA treatment, and later studies revealed that unlike wild-type controls, STC-1 knockout mice were unresponsive to IOP reduction when treated with LFA. Furthermore, topical application of recombinant STC-1 protein lowered IOP in normotensive [38, 39] and ocular hypertensive [40] mice. Finally, topically administered STC-1 showed equivalent IOP reduction as topically administered LFA, but STC-1 did not require the FP receptor for IOP reduction [39]. Given these unique features, we hypothesized that transgene expression of STC-1 in the anterior chamber of the mouse eye would provide sustained IOP reduction.

## Materials and methods

### Mouse experiments

All animal studies and treatment protocols were pre-approved by the Mayo Clinic (Rochester, MN) Institutional Animal Care and Use Committee and adhered to the ARVO Statement for the Use of Animals in Ophthalmic and Vision Research, Basal Declaration, and the International Council for Laboratory Animal Science. Furthermore, the ARRIVE guidelines to ensure reliability of research reporting were followed. All mouse strains had unrestricted access to food and water and were housed with 12 hour alternating light and dark cycles. Mice were humanely euthanized using carbon dioxide asphyxiation followed by cervical dislocation.

### Adeno-associated viral vector

Single strand (ss) adeno-associated virus (AAV) serotype 2 (AAV2) to express STC-1 with a FLAG tag (ssAAV2-STC-1-FLAG) was generated as previously described [41]. The construct was designed based on prior neuroprotection studies performed by our laboratory using ssAAV2-STC-1-FLAG containing a human SYN1 promoter, specific for retinal ganglion cells [42]. For the current studies, we replaced the human SYN1 promoter with the chicken β-actin (CBA) promotor to allow for ubiquitous ocular expression following an intracameral injection. The vector was packaged in a capsid-modified AAV2 vector containing 3 surface-exposed tyrosine to phenylalanine mutations (triple Y-F). For a control, an identical vector was generated expressing green fluorescent protein (GFP; ssAAV2-GFP) in place of the STC-1-FLAG transgene.

### Intracameral injection

Three-month-old C57BL/6J wild-type or FP receptor knockout mice previously generated by our laboratory [39] were anesthetized with an intraperitoneal injection of ketamine/xylazine/acepromazine (80/6/1 mg/kg body weight) for intracameral injections. Mice were subsequently placed on a dissecting microscope to visualize the anterior chamber. A 32 gauge beveled needle (Hamilton Company, Reno, NV) containing the AAV2 construct (1 μL; 3 x 10^12^ VG/mL) or 1 μL phosphate buffered saline (PBS) was placed parallel to the cornea and inserted anterior to the limbus into the anterior chamber. Care was taken to avoid iris trauma as well as avoidance of the corneal endothelium and anterior lens capsule. The injected volume was administered into the anterior chamber over 2 seconds. The needle was then slowly removed to minimize reflux.

### IOP measurements

IOP was measured in conscious mice using a handheld rebound tonometer (Icare TonoLab; Colonial Medical Supply, Franconia, NH). For measurements of IOP, the conscious mouse was gently restrained in a decapicone (Braintree Scientific, Inc., Braintree, MA) and the probe of the Tonolab tonometer was positioned perpendicular and approximately 3 mm from the mouse cornea. Upon initiation, the probe extends out and rebounds off the cornea six times, each time taking a measurement. The internal software discards the highest and lowest values and shows the average of the remaining four values as a single measurement. For each IOP measurement, three sequential but independent readings were obtained, averaged, and recorded. A single laboratory technician measured and recorded all IOPs while a second laboratory member who was masked to treatment groups checked IOP at multiple points throughout the experiment to ensure accurate data collection.

For all experimental protocols, mice underwent 3–4 days of twice daily Tonolab measurements to obtain baseline IOP prior to treatment. These measurements were averaged and taken as a single baseline value. After treatment, IOPs were measured twice daily up to four times weekly for the duration of each experiment. For summary graphs and statistical analysis, IOP was averaged, taken as a single value, and presented as weekly, bi-monthly, or monthly values.

### Assessment of STC-1-FLAG protein expression

To determine site of expression of STC-1-FLAG at the protein level, immunohistochemistry was performed. Mice were euthanized, whole globes were enucleated, fixed in 4% paraformaldehyde in 0.1M phosphate buffer, and embedded in paraffin. Paraffin blocks were sectioned at 5 μm and mounted on poly-L-lysine coated slides. Following deparaffinization, antigen retrieval was performed by placing slides in a 1 mM solution of EDTA pH 8.0 at 95°C for 30 min. Slides were rinsed twice at room temperature for five minutes each in PBS. Slides were placed in a humidified chamber and sections were blocked with 10% goat serum and 3% albumin in PBS at room temperature for 30 minutes. Following incubation, slides were rinsed with PBS at room temperature twice for 2 minutes. Sections were incubated with anti-FLAG primary antibody (1:100; MilliporeSigma, St. Louis, MO) in 1% ovalbumin overnight at 4°C. Slides were rinsed twice in PBS at room temperature for 5 minutes and then incubated with Alexa Fluor 488 antibody (1:200; Abcam) for one hour at room temperature. Vectashield containing DAPI (Vector Laboratories, Burlingame, CA) was added to the slides, followed by placement of a coverslip, and imaged on a Zeiss LSM780 confocal microscope (Zeiss, Dublin, CA).

### Evaluation of RNA expression by ssAAV2-STC-1-FLAG

To determine expression of ssAAV2-STC-1-FLAG at the gene level, mice were euthanized and whole globes were enucleated by surgical excision and immediately placed in RNA isolation reagent (RNA Bee; Tel-Test Inc, Friendswood, TX) and frozen at -80°C. Prior to RNA isolation, samples were removed from the freezer, thawed, and homogenized on ice. Following removal of the aqueous phase, total RNA was isolated and purified (RNeasy Mini kit; Qiagen, Valencia, CA). cDNA was generated by reverse transcription using 1 μg total RNA (iScript cDNA synthesis kit, Bio-Rad Laboratories, Hercules, CA). Oligonucleotides were designed to amplify STC-1-FLAG avoiding amplification of endogenous STC-1 transcripts by designing the forward primer in the STC-1 sequence and the reverse primer in the FLAG tag sequence (Forward primer: 5’-GTGCTTCTGCAACCCATGAGG-3’; Reverse primer: 5’-CTTGTCATCGTCGTCCTTGTAGTCG-3’). PCR reactions were prepared, and the following PCR conditions were used: 95°C, 6 min, 1 cycle; 95°C, 1 min, 60°C, 1 min, 68°C, 2.5 min, 40 cycles; 68°C, 7 min, 1 cycle; 4°C until recovery, 1 cycle. Aliquots of each PCR reaction (15 μL) were separated on a 1% agarose gel.

### Assessment of aqueous outflow parameters by constant flow infusion

To determine the effects of ssAAV2-STC-1-FLAG on parameters of aqueous outflow, 3-month-old C57BL/6J mice were separately treated with the following: 1) intracameral ssAAV-STC-1-FLAG (n = 5), 2) intracameral ssAAV2-GFP (n = 5), 3) topical recombinant human STC-1-FLAG (n = 4; Biovender Research and Diagnostic Products, Czech Republic) [38, 39], 4) topical LFA (n = 5; Cayman Chemical, Ann Arbor, MI), or 5) untreated mice (n = 4). Mice that received topical treatments were treated with once daily topical doses of STC-1 (5 μl of a 0.5 μg/μl solution) or LFA (10^−4^ M) for 5 days prior to aqueous outflow experiments. Mice that received intracameral injections were treated 6 weeks prior to aqueous outflow measurements. Aqueous outflow was assessed using the protocol established by Millar et al. [43] and previously used by our laboratory [44]. Briefly, a 33 gauge needle was used to cannulate the anterior chamber of anesthetized mice. The needle was connected to a flow-through pressure transducer (World Precision Instruments, Sarasota, FL) that was also connected to an SP101i microdialysis infusion pump (World Precision Instruments) and an open-ended, variable-height, raised reservoir manometer. Episcleral venous pressure was obtained by visualizing the reflux of blood into Schlemm’s canal by direct ophthalmoscopy as the reservoir was lowered. Outflow facility was determined by slowly increasing the flow rate with the infusion pump and recording the pressure. Uveoscleral outflow was calculated when the inflow was presumed to be zero immediately after animal sacrifice. Aqueous humor formation rate was then estimated by using the modified Goldmann equation: aqueous humor formation rate = [outflow facility X (IOP–episcleral venous pressure)] + uveoscleral outflow [43]. To validate the calculated IOP values based on the Goldmann equation, IOP was also assessed with a handheld rebound tonometer. All data were recorded and analyzed using LabScribe software (World Precision Instruments).

### Statistics

Students t-test was used to compare treatment groups to controls for all experiments when a single comparison was made. For aqueous humor dynamics experiments, an ANOVA with a Bonferroni post-hoc analysis was used to control for multiple variables Values expressed as mean ± standard deviation, and values <0.05 were considered to be significant.

## Results

### The early IOP lowering response following injection with ssAAV2-STC-1-FLAG

**Fig 1:**
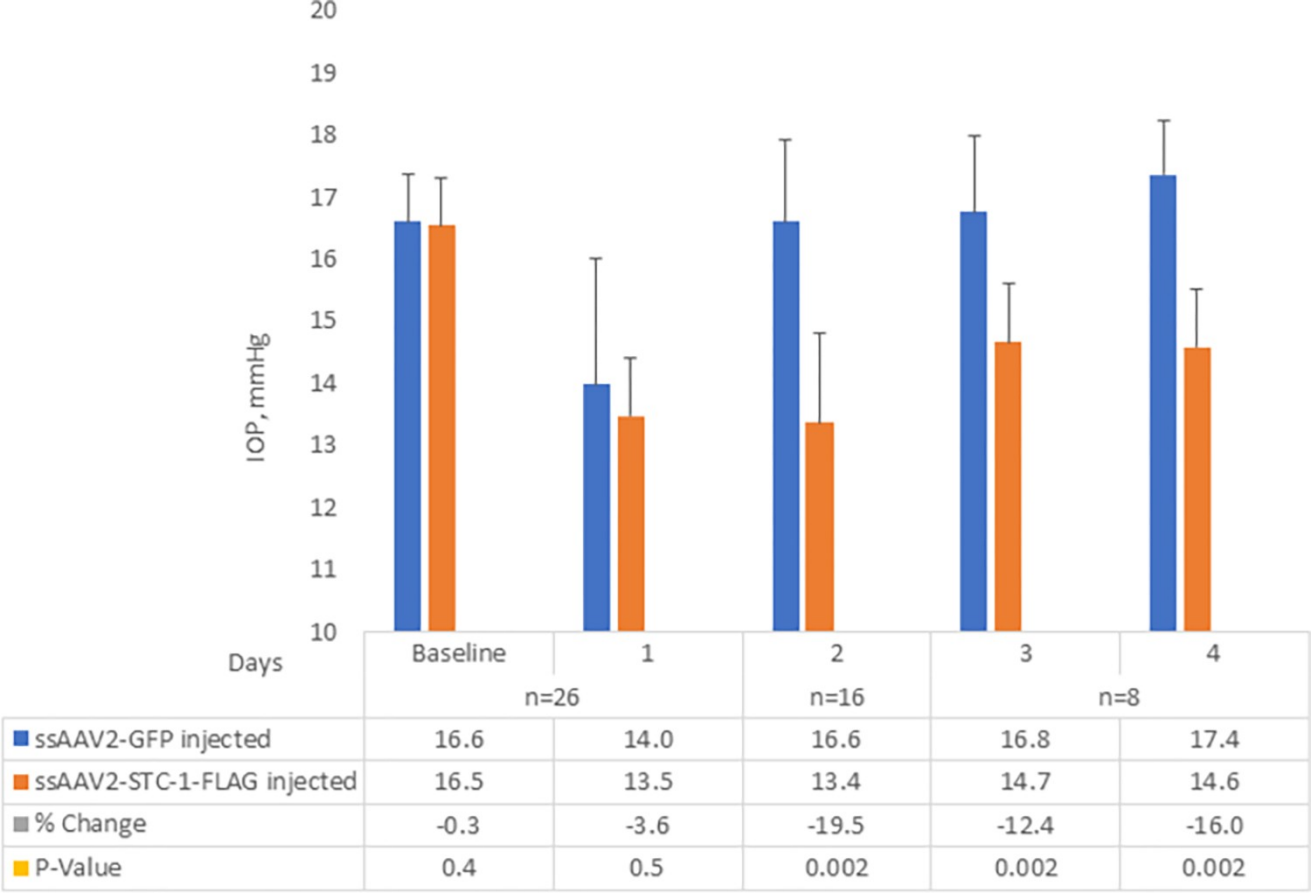
Early IOP lowering response following injection with ssAAV2-STC-1-FLAG. After baseline IOP measurements, three-month-old C57BL/6J mice (n = 26) received a single intracameral injection of ssAAV2-STC-1-FLAG in one eye and ssAAV2-GFP in the fellow eye (1 μL, intracameral injection, 3x10^9^ VGs). After an initial, non-significant decrease in IOP in both ssAAV2-STC-1-FLAG treated mice and ssAAV2-GFP control mice at day 1, and a return to baseline with ssAAV2-GFP by day 2, significant IOP lowering was seen following treatment with ssAAV2-STC-1-FLAG starting at day 2. This IOP reduction persisted until the end of the experiment at day 4.

**Fig 2:**
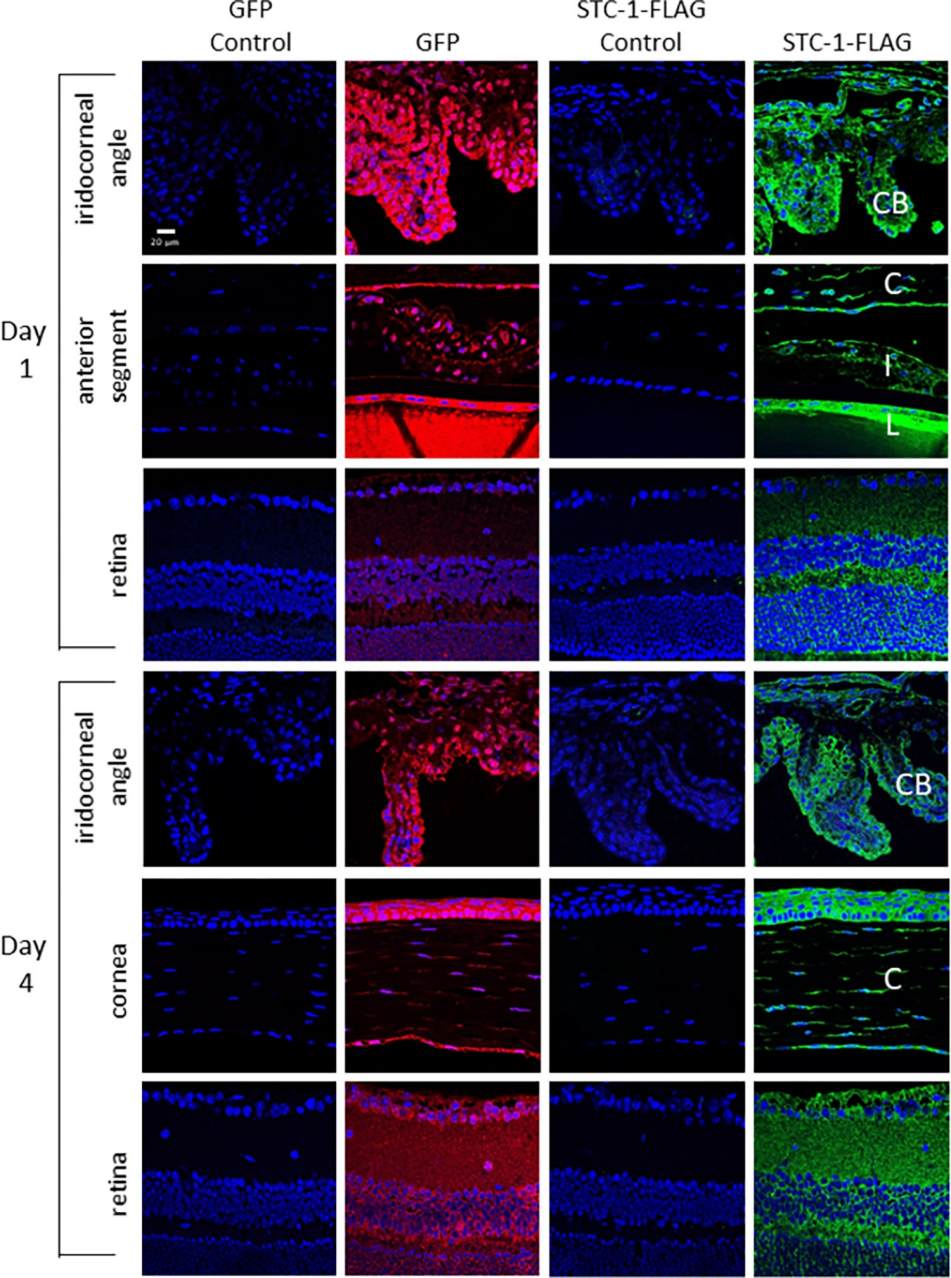
ssAAV2-STC-1-FLAG shows diffuse intraocular expression after intracameral injection. Three-month-old C57BL/6J mice (n = 26) received a single intracameral injection of ssAAV2-STC-1-FLAG in one eye and ssAAV2-GFP in the fellow eye (1 μL, intracameral injection, 3x10^9^ VGs). Diffuse ocular expression was seen following delivery of STC-1-FLAG or GFP with the AAV2 vector. Cells in the iridocorneal angle including ciliary body (CB), anterior segment including cornea (C), iris (I), and lens capsule (L), and the retina showed expression of GFP (red fluorochrome) and STC-1-FLAG (green fluorochrome) at day one post-injection with ssAAV2-GFP and ssAAV2-STC-1-FLAG. Similar results were seen 4 days post-injection.

In order to determine whether STC-1-FLAG expressed by an AAV lowered IOP, C57BL/6J mice with no difference in baseline IOP between fellow eyes (16.5 ± 0.8 mmHg vs 16.6 ± 0.8 mmHg, P = 0.4, n = 26, Fig 1) received a single intracameral injection of ssAAV2-STC-1-FLAG (3 x 10^9^ VGs) in one eye and ssAAV2-GFP (3 x 10^9^ VGs) in the fellow eye. IOP was assessed daily, and tissues were collected at days 1 and 4 for expression analysis. At day one post-injection, there was no significant difference in IOP between ssAAV2-STC-1-FLAG and ssAAV2-GFP-injected animals (13.5 ± 0.9 vs. 14.0 ± 2.0 mmHg, P = 0.5, n = 26, Fig 1), although both eyes showed an IOP drop from baseline. On day 2, IOP in the eyes injected with ssAAV2-GFP had returned to baseline levels (16.6 ± 1.3 mmHg), but the eyes injected with ssAAV2-STC-1-FLAG remained lower (13.4 ± 1.4 mmHg) showing a significant change (P<0.01, n = 16, Fig 1). Immunohistochemistry revealed GFP fluorescence (red fluorochrome) as well as STC-1-FLAG expression (green fluorochrome) starting at day 1 in the iridocorneal angle with notable expression in the ciliary body, in the anterior segment with expression in the cornea, iris, and lens capsule, and in the retina (Fig 2). Expression in these tissues remained evident through termination of the experiment at day 4 (Fig 2).

### ssAAV-2-STC-1-FLAG reduces IOP in a sustained manner

**Fig 3:**
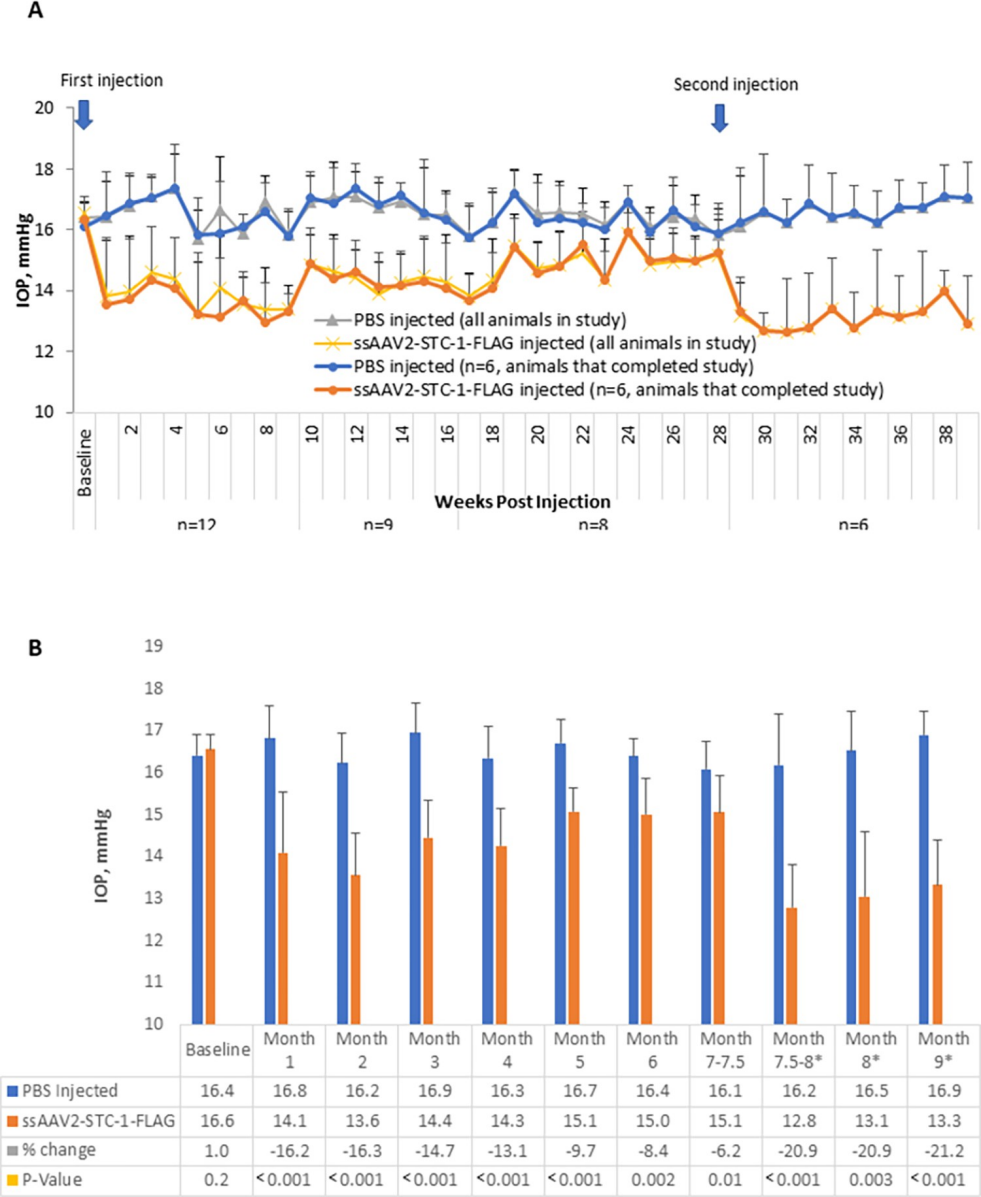
ssAAV2-STC-1-FLAG reduces IOP in a sustained fashion. A) After baseline IOP measurements, ssAAV2-STC-1-FLAG was injected in one eye and PBS was injected into the fellow eye of 3-month-old C57BL/6J mice (n = 12, 1 μL, intracameral injection, 3x10^9^ VGs). At the end of experimental week 28, all remaining mice in the study (n = 6) received a second injection. IOP reduction was observed by week one post-injection and this persisted through the end of the experimental period. As IOP lowering began to wane, this was rescued with a repeat injection at week 28. Animals that completed the study were separately designated to demonstrate representative samples. B) Similar data presented in a bar graph format with statistical analysis performed on the average monthly values. *Data where mice had received a second injection.

In order to determine whether STC-1 expression in the anterior chamber results in IOP reduction in a sustained manner, C57BL/6J mice with no difference in baseline IOP between fellow eyes (16.4 ± 0.5 vs 16.6 ± 0.4 mmHg, P = 0.2, n = 12, Fig 3A and 3B), received a single intracameral injection of ssAAV2-STC-1-FLAG (3 x10^9^ VGs) in one eye and an intracameral injection of PBS in the fellow eye. Averaged weekly IOP measurements showed IOP reduction in ssAAV2-STC-1-FLAG injected eyes compared to PBS injected fellow eyes from week 1 through week 28 following the single intracameral injection (Fig 3A). For the first 4 months, IOP in the ssAAV2-STC-1-FLAG injected eye was lower compared to the contralateral eye by 13.1–16.3% (>2.1 mmHg). In months 5 and 6, the IOP reduction was slightly less with percent change of 8.4–9.7% (>1.3 mmHg). Statistically, IOP reduction remained significant from month 1 with a 16.2% reduction (16.8 ± 0.8 vs 14.1 ± 1.4 mmHg, P<0.001, n = 12, Fig 3B) to month 7 with a 6.2% reduction (16.1 ± 0.6 vs 15.1 ± 0.9 mmHg, P = 0.01, n = 8, Fig 3B). Though the average IOP reduction at month 7 was less than 50% of the IOP reduction seen at 1 month, the difference was not statistically significant (14.1 vs 15.0 mmHg, P = 0.2). Note, at week 9, three animals were selected at random for sacrifice for attempted expression analysis, but results were not obtained due to a technical error and one animal died of unknown causes at week 16.

**Fig 4:**
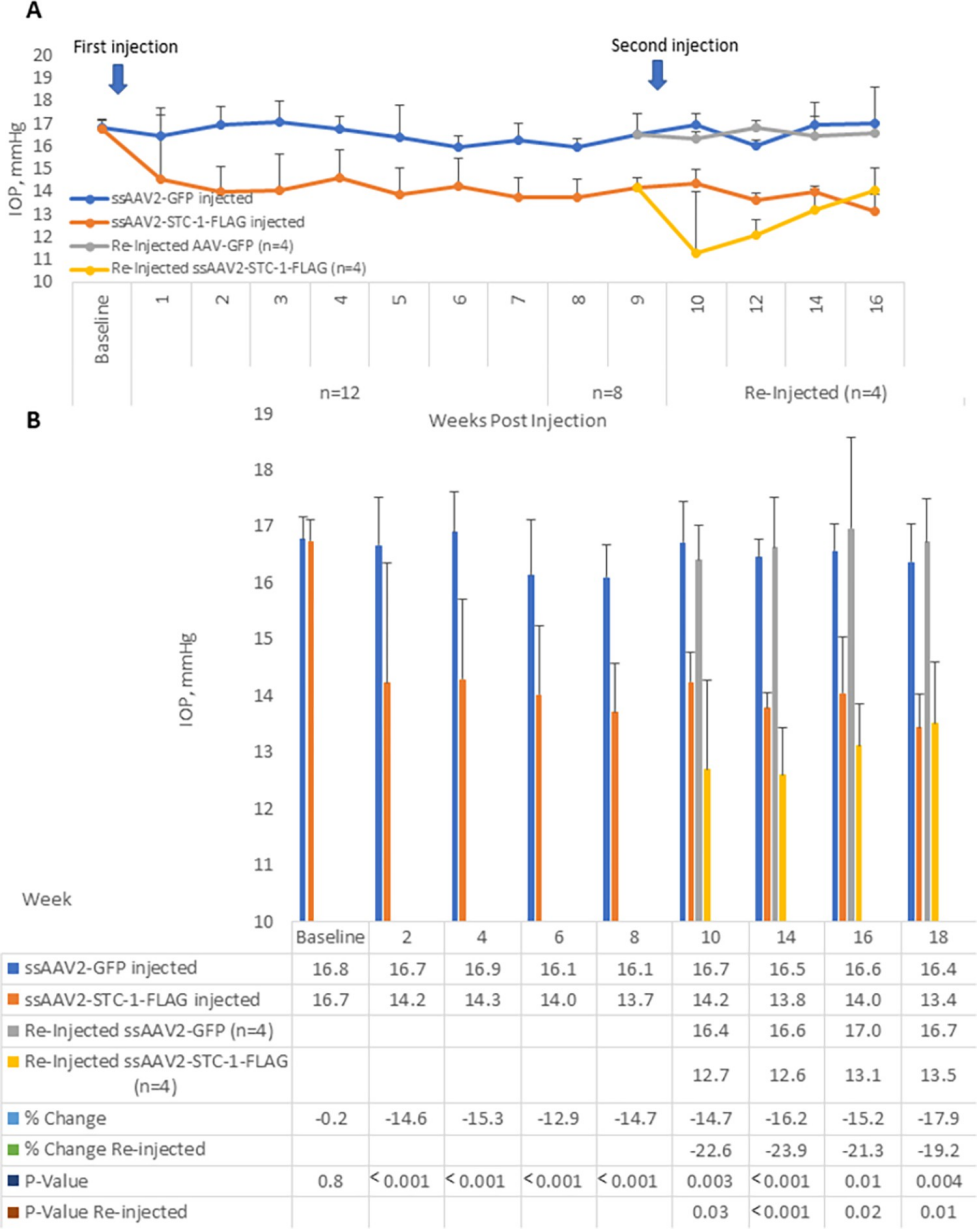
ssAAV2-STC-1-FLAG reduces IOP compared to ssAAV2-GFP. A) After baseline IOP measurements, ssAAV2-STC-1-FLAG (3x10^9^ VGs) was injected in one eye and ssAAV2-GFP (3x10^9^ VGs) was injected into the fellow eye (n = 22) of 3-month-old C57BL/6J mice. IOP was lowered by week 1. At the end of week 8, remaining mice in the study (n = 8) were randomized to continue without further treatment (n = 4) or receive a second injection (n = 4). By the end of week 16, there was no detectable difference in IOP lowering between once-injected and twice-injected ssAAV2-STC-1-FLAG treated mice. B) Similar data presented in a bar graph format with statistical analysis performed bi-monthly post-injection.

To determine whether a second injection would restore the IOP reduction observed within the first 4 months, eight animals were re-injected with a second dose of ssAAV2-STC-1-FLAG in the treated eye and PBS in the fellow control eye at 7.5 months following the initial injection. Two animals did not survive repeat anesthesia. One week following the second injection, IOP was 20.9% lower in eyes re-injected with ssAAV2-STC-1-FLAG compared to fellow PBS re-injected control eyes (13.1 ± 1.5 vs. 16.5 ± 0.9 mmHg, P = 0.0002, n = 6, Fig 3B) and remained lower for 2 additional months when the experiment was stopped to collect tissues for transgene expression analysis. Assessment of IOPs in animals at month 8 (second injection) compared to month 7 (single injection) showed that following the second injection of AAV-STC-1-FLAG, eyes had a significantly lower IOP (13.1 ± 1.5 vs. 15.1 ± 0.9 mmHg, P<0.05). To determine whether the initial IOP lowering was superior following two injections compared to one injection, month 8 ssAAV-STC-1-FLAG twice-injected eyes were compared to once-injected ssAAV2-STC-1-FLAG eyes at month 1 and no significant difference was seen (13.1 ± 1.5 vs 14.1 ± 1.4 mmHg, P = 0.5). Fig 4A contains a separate line graph showing the six re-injected animals are a representative sample of the original cohort.

To assess STC-1-FLAG transgene expression, ocular tissues were collected from the remaining animals sacrificed at the completion of the experiment (week 38). Mice that had received two intracameral injections of ssAAV2-STC-1-FLAG showed expression of the STC-1-FLAG transgene at the expected size of 728 base pairs (S1 Fig).

### ssAAV2-STC-1-FLAG reduces IOP in a sustained manner compared to ssAAV2-GFP

In order to correlate tissue expression with repeat injections of ssAAV2-STC-1-FLAG, C57BL/6J mice with no difference in baseline IOP between fellow eyes (16.6 ± 0.4 vs 16.5 ± 0.4 mmHg, P = 0.4, n = 12, Fig 4A and 4B) received an intracameral injection of ssAAV2-STC-1-FLAG (3x10^9^ VGs) in one eye and an intracameral injection of ssAAV2-GFP (3x10^9^ VGs) in the fellow eye. IOP was assessed starting 4 days post-injection. Eyes that received ssAAV2-GFP showed no major change in IOP after injection throughout the course of the experiment (Fig 4A, 4B). In contrast, eyes that received ssAAV2-STC-1-FLAG showed consistent IOP reduction over the same time interval. At week 2 post-injection, IOP was lowered an average of 14.6% in ssAAV2-STC-1-FLAG injected eyes compared to fellow ssAAV2-GFP injected eyes (14.2 ± 2.1 vs 16.7 ± 0.8 mmHg, n = 12, P<0.001, Fig 4B). Four animals were euthanized at week 8 for expression analysis. To address whether additional IOP-reduction could be obtained with a repeat injection, at week 9 half of the remaining 8 animals were randomized to a second injection (n = 4). At week 10 of the experimental protocol (one week after the second injection), IOP was lowered 22.6% in ssAAV2-STC-1-FLAG twice injected eyes compared to ssAAV2-GFP twice injected eyes (12.7 ± 1.6 vs. 16.4 ± 1.6 mmHg, P<0.001, Fig 4B). This trend continued until the completion of the experiment at week 18 when all remaining animals were euthanized for expression analysis.

When comparing eyes that had received a single injection of ssAAV2-STC-1-FLAG with eyes that received a second injection, both at week 10, the values were not statistically different (14.2 ± 0.5 vs 12.7 ± 1.6 mmHg, n = 4, P = 0.1, Fig 4B). To determine whether IOP was lower one month after the second injection compared to 1 month after the first injection, week 14 twice injected ssAAV2-STC-1-FLAG eyes were compared to week 4 once injected ssAAV2-STC-1-FLAG eyes. Though there was a trend toward lower IOP (12.6 ± 0.8 vs 14.3 ± 1.4 mmHg, P = 0.1, Fig 4B), the results were not statistically significant.

**Fig 5:**
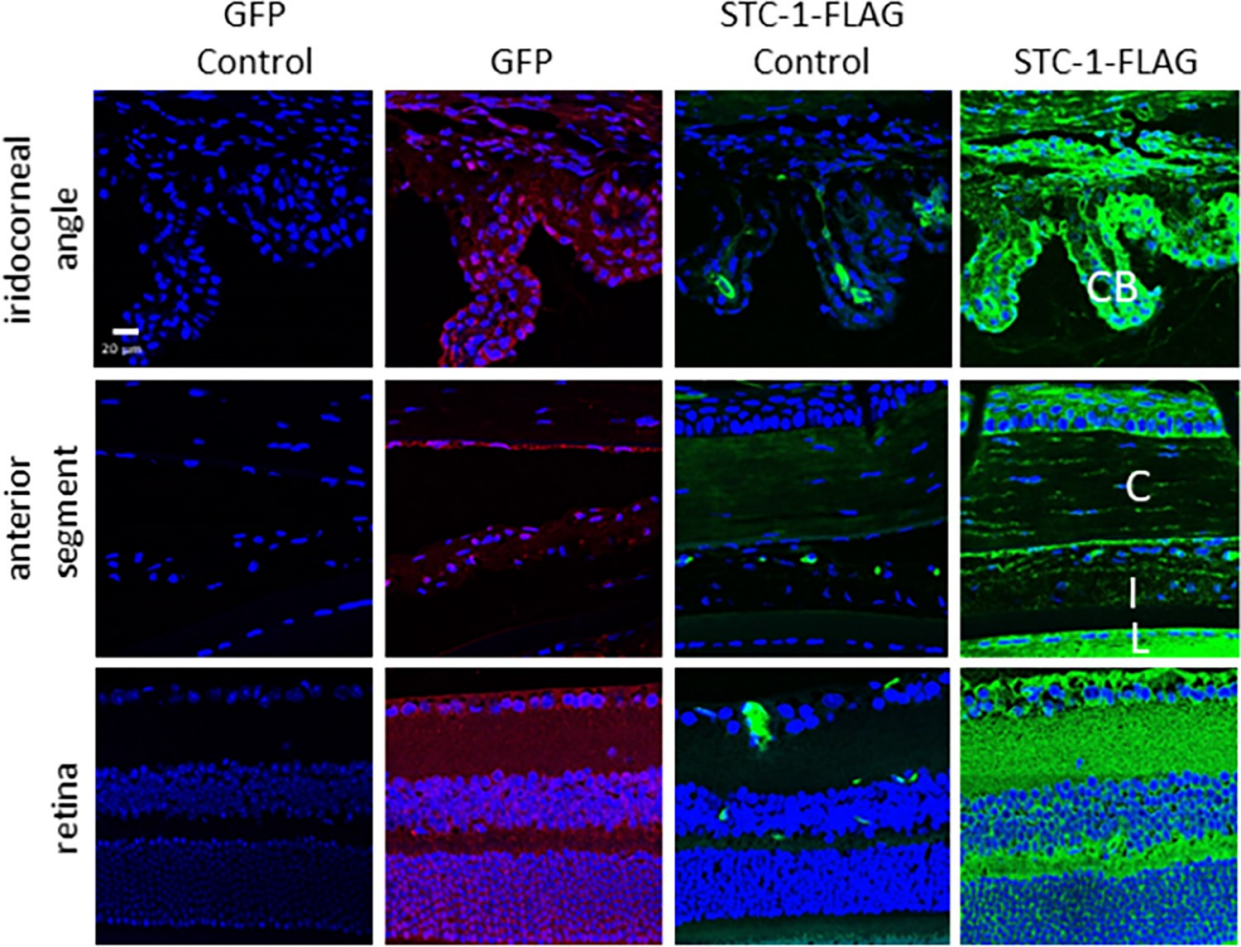
ssAAV2-STC-1 is expressed in a sustained fashion after intracameral injection. ssAAV2-STC-1-FLAG (3x10^9^ VGs) was injected in one eye and ssAAV2-GFP (3x10^9^ VGs) was injected into the fellow eye (n = 22) of 3-month-old C57BL/6J mice. At 8 weeks post-injection, diffuse intraocular STC-1-FLAG expression was seen in the iridocorneal angle including ciliary body (CB); the anterior segment including cornea (C), iris (I), and lens capsule (L); and retina. Note mild autofluorescence in STC-1-FLAG controls.

Eyes that received an intracameral injection of ssAAV2-STC-1-FLAG showed STC-1-FLAG expression in the ciliary body, corneal endothelium, lens epithelium, iris, and retina (green fluorochrome) at week 8 (Fig 5). Similar patterns of expression were seen at week 18 after a single intracameral injection and at week 18 after a second injection at week 10 (S2 Fig). Eyes that were injected with ssAAV2-GFP (red fluorochrome) showed a similar pattern of expression to ssAAV2-STC-1-FLAG in that expression could be detected in the ciliary body, corneal endothelium, lens epithelium, iris, and retina (S2 Fig).

### ssAAV2-STC-1-FLAG reduces IOP independent of the FP receptor

**Fig 6:**
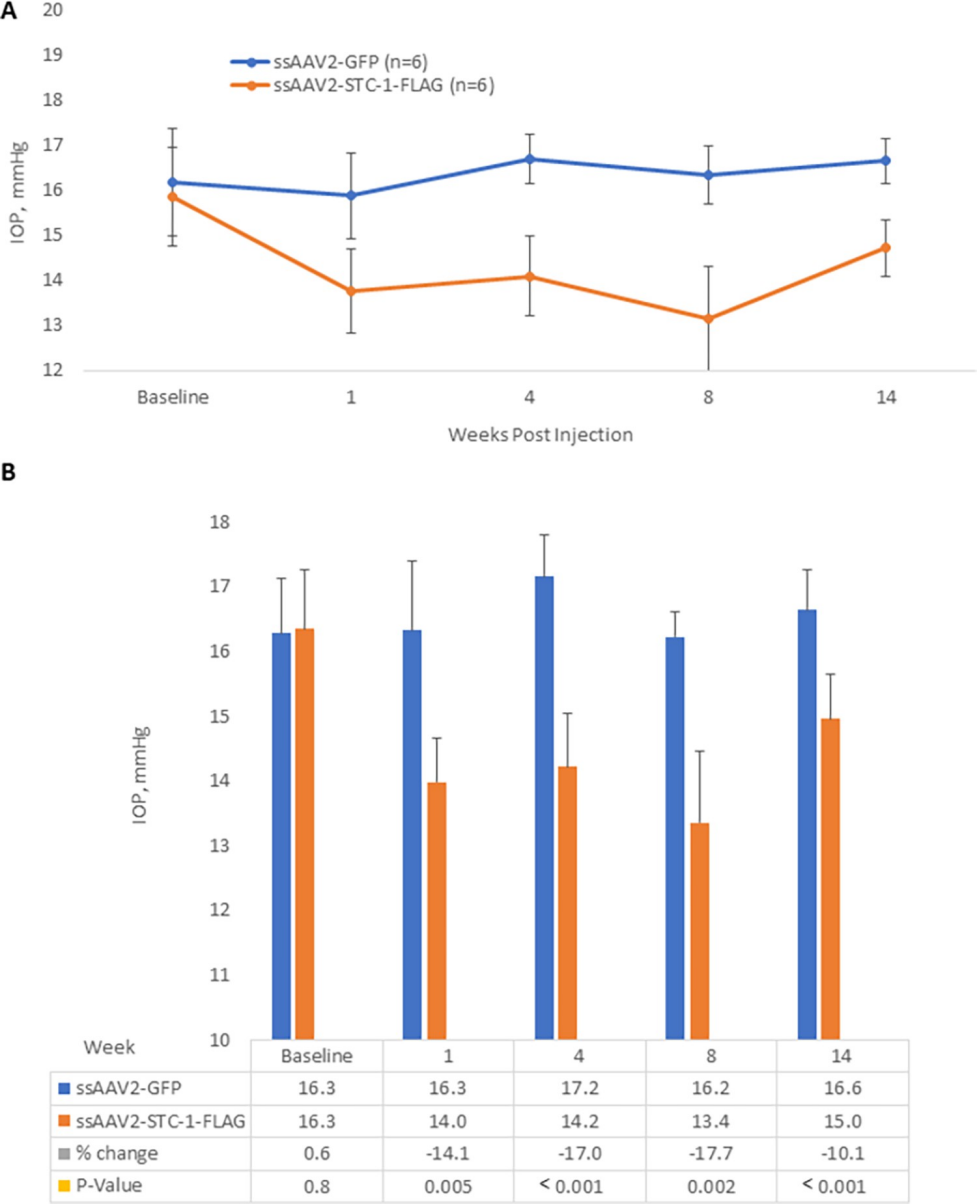
ssAAV2-STC-1-FLAG reduces IOP in the FP receptor knockout mouse. A) After baseline IOP measurements, ssAAV2-STC-1-FLAG (3x10^9^ VGs) was injected in one eye and ssAAV2-GFP (3x10^9^ VGs) was injected into the fellow eye (n = 6) of 3-month-old FP receptor knockout mice. IOP lowering was seen in mice treated with intracameral ssAAV2-STC-1-FLAG starting by week 1 post-injection and this persisted through the remainder of the experiment indicating that IOP-lowering is independent of the FP receptor. B) Data presented in a bar graph with statistical analysis performed at week 1, 4, 8, and 14 post-injection.

The ability of latanoprost to lower IOP depends on its binding to the FP receptor and activation of downstream signaling cascades [45]. We had previously shown that STC-1 was necessary for latanoprost-mediated IOP reduction [38] and when applied topically as an eye drop, could lower IOP through an FP receptor independent mechanism [39] To determine whether sustained IOP reduction following ssAAV2-STC-1-FLAG injection also did not require the FP receptor, we performed intracameral injections of ssAAV2-STC-1-FLAG in one eye and ssAAV2-GFP in the fellow eye of FP receptor knockout mice previously generated by our laboratory [28]. There was no difference in baseline IOP measurements between fellow eyes (16.3 ± 0.8 vs 16.3 ± 0.9 mmHg, n = 6, P = 0.8, Fig 6A). After injection, compared to fellow control eyes, IOP was reduced in FP receptor knockout mice injected with ssAAV2-STC-1-FLAG by 15.5% (16.4 ± 1.1 vs 14.1 ± 0.7 mmHg, n = 6, P<0.001, Fig 6A and 6B) at week 4 and by 19.4% (16.2 ± 0.4 vs 13.5 ± 1.1 mmHg, n = 6 P<0.001, Fig 6A and 6B) at week 8. This suggests that sustained IOP reduction with STC-1-FLAG expression is not dependent on the FP receptor.

### ssAAV2-STC-1-FLAG reduces IOP by increasing outflow facility

**Fig 7:**
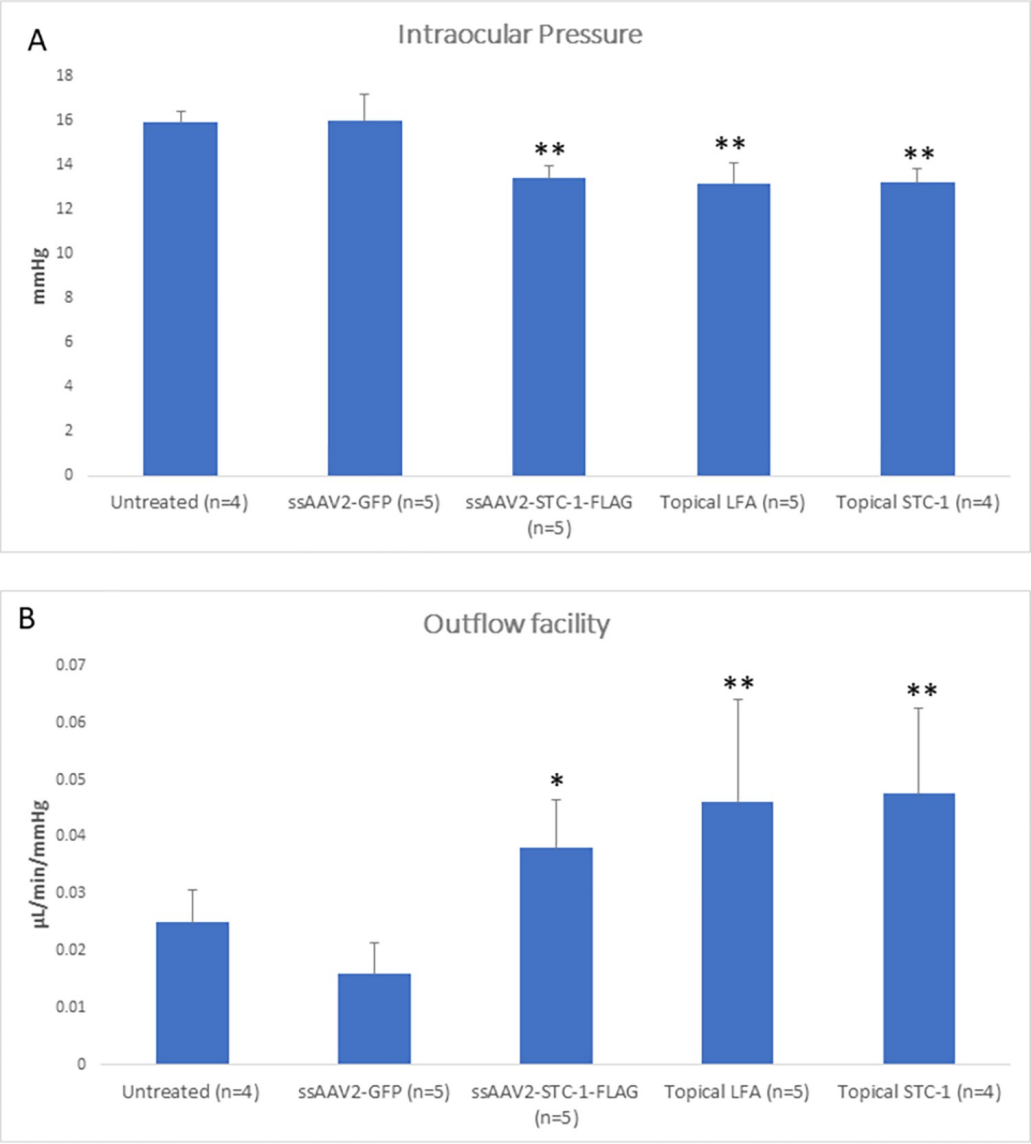
ssAAV2-STC-1-FLAG reduces IOP by increasing outflow facility. 3-month-old C57BL/6J mice were randomized to receive either a single injection of intracameral ssAAV2-STC-1-FLAG (n = 5; 3x10^9^ VGs) or ssAAV2-GFP (n = 5; 3x10^9^ VGs) or daily topical STC-1-FLAG (n = 4), LFA (n = 5) or no treatment (n = 4) in one eye. IOP was measured, and aqueous humor dynamics was performed at maximal treatment response (week 6 for injection experiments and day 5 for topical experiments). A) IOP was significantly reduced in mice treated with intracameral ssAAV2-STC-1-FLAG, topical LFA, and topical STC-1-FLAG compared to intracameral ssAAV2-GFP. No significant difference was seen between untreated and ssAAV2-GFP-injected mice. B) Outflow facility was significantly increased in mice treated with intracameral ssAAV2-STC-1-FLAG, topical LFA, and topical STC-1-FLAG compared to intracameral ssAAV2-GFP. No significant difference was seen when comparing untreated and ssAAV2-GFP-injected mice. *P<0.05, ** P<0.01.

In order to determine the mechanism of action of IOP lowering by ssAAV2-STC-1-FLAG, aqueous outflow measurements were performed. Three-month-old C57BL/6J mice (n = 14) were randomized to treatment with a single injection of intracameral ssAAV2-STC-1-FLAG (n = 5), a single injection of intracameral ssAAV2-GFP (n = 5), or a no treatment control (n = 4). In addition, a separate cohort of age-matched C57BL/6J mice (n = 9) were randomized to treatment with daily topical administration of LFA (n = 5) or human recombinant STC-1-FLAG (n = 4). At maximal IOP reduction (6 weeks post-injection for intracameral injections and treatment day 5 for topical treatments), aqueous humor dynamic studies by perfusion were performed. There was a significant difference among treatments with respect to IOP (P<0.001) with ssAAV2-STC-1-FLAG (P<0.001), topical LFA (P<0.001), and topical STC-1-FLAG (P<0.001) each having significantly reduced IOP compared to AAV2-GFP (Fig 7A). A significant increase in outflow facility was also observed among treatments (P = 0.002) with ssAAV2-STC-1-FLAG (P<0.05), topical LFA (P<0.001), and topical STC-1-FLAG (P<0.001) when compared to ssAAV2-GFP (Fig 7B). There was no significant difference in EVP (P = 0.2, S3A Fig) or uveoscleral outflow (P = 0.2, S3B Fig), or aqueous humor formation rate (P = 0.5, S3C Fig) among treatment groups.

## Discussion

Lowering of IOP remains the only reliable therapeutic target for the 80 million people worldwide afflicted by GON [2]. Therapeutics that provide sustained IOP reduction have the potential to revolutionize glaucoma treatment by limiting the compliance issues that keep half of glaucoma patients from taking medications as prescribed [16–20]. In the current study, we found that diffuse and sustained expression of STC-1-FLAG by way of AAV2 delivery reduced IOP for up to 6 months following a single injection. Furthermore, as the IOP-lowering effect began to wane, full IOP-lowering could be achieved with a repeat injection. In mice, the mechanism of IOP reduction was by increasing outflow facility similar to that of both topical STC-1-FLAG and topical LFA. However, unlike LFA, ssAAV2-STC-1-FLAG did not require the FP receptor for IOP reduction.

Similar to previous reports [46], our data show minimal IOP change from baseline ssAAV2-GFP injected fellow eye controls following intracameral injection with the exception of a non-significant drop in IOP day one after injection. This indicates that the ssAAV2 vector does not have significant IOP altering effects by itself similar to an injection with PBS alone. Transgene expression of STC-1-FLAG was detected day one after intracameral injection and IOP reduction was observed starting on day 2. A 16% IOP reduction was maintained for the first two months after injection, similar to what we have previously reported with daily topical treatment with either LFA or recombinant STC-1-FLAG protein [39]. By 6 months post-injection of ssAAV-STC-1-FLAG, the percent IOP lowering had diminished 50% but the effect was still statistically significant compared to controls. Repeat treatment with ssAAV2-STC-1-FLAG was sufficient to produce the full IOP reduction as seen with the first injection. This is a significant finding as sustained IOP reduction that can be maintained with repeat injections has important therapeutic implications for patients that suffer the burden of daily topical IOP-lowering treatments. Repeat intraocular injections have become the mainstay of treatment for a number of ocular conditions. For example, intraocular anti-VEGF agents are used to treat a number of retinal diseases including macular degeneration, diabetic retinopathy, and retinal vascular occlusions with some patients receiving, and tolerating well, monthly injections for years [47]. This observation has highlighted the feasibility of intraocular injections of a number of therapeutic compounds, including gene therapy, for several ocular conditions including GON or ocular hypertension. It should be noted, however, that the long-term effects of multiple repeated AAV injections, including potential host immune response, in animal models and humans are unknown.

The ssAAV2 vector produced intraocular expression of both GFP and STC-1-FLAG. GFP expression was evident by day one post-injection similar to prior reports [48]. STC-1-FLAG expression was also detectable by day one post-injection and continued to be expressed at all time points. It is interesting to note that STC-1-FLAG expression levels at week 8 post-injection appeared higher by immunohistochemistry compared to week 18. We also saw stronger STC-1-FLAG signal following a second injection compared to those that had received only one injection. The pattern of STC-1-FLAG expression appeared to correlate with the level of IOP reduction and may explain the slow drop in IOP that can be subsequently rescued with a repeat injection. However, our analysis is subjective at this point as it is difficult to obtain reliable and accurate quantitative expression data with immunohistochemistry, particularly with small samples sizes. Further studies are needed to determine how STC-1-FLAG expression changes over time and correlate this with IOP reduction.

Gene therapy for glaucoma has been limited since a single identified gene defect is rare for patients with GON [49]. Use of viral vectors to modify extracellular matrix remodeling or to impact the PGF2α/FP receptor pathway have shown minimal success [50–52]. However, Luna et al. recently reported delivery to the anterior chamber of AAV2 carrying the transgene for miR-146a, a microRNA that is involved in the regulation of fibrosis and inflammation [53], resulting in sustained IOP reduction. In our current study, overexpression of a naturally occurring, IOP lowering protein rather than gene editing provides an opportunity to apply treatment broadly for all types of GON or ocular hypertension.

AAVs are currently the vector of choice for intraocular gene therapy due to their efficacy and safety profile [54]. Furthermore, proteins delivered by AAV viral vectors (i.e. green fluorescent protein) have shown sustained expression in the trabecular meshwork for over two years in non-human primates, making AAV an attractive construct for delivery to the anterior chamber [55]. Of the available constructs, AAV2 has the most extensive track record and safety profile for intraocular gene therapy [56] including in clinical trials, where they have been used to deliver RPE65 in Leber congenital amaurosis [57]. Success of transgene expression includes both cellular entry, transfectivity, and cell specificity. For example, single-stranded vector packaging of AAV2 has robust entry but minimal transfectivity of trabecular meshwork unless self-complementation is used [58]. Though the trabecular meshwork is an important tissue involved in aqueous humor drainage, expression of STC-1 by trabecular meshwork cells is not required since STC-1 is a secreted peptide hormone with autocrine, intracrine, and paracrine functions [59]. Furthermore, the natural fluid dynamics of the anterior chamber will deliver viral vectors and/or secreted proteins into the natural outflow pathways of the eye [60]. Therefore, expression of STC-1-FLAG via any anterior chamber tissue may provide sufficient amounts of secreted STC-1 necessary for IOP reduction. In our study, we observed FLAG-tagged STC-1 in the ciliary body, cornea, iris, lens, and retina. In addition to IOP reduction, STC-1-FLAG expression in the retina following intracameral injection has important implications for direct neuroprotective effects of retinal ganglion cells given its previously described retinal neuroprotective functions [28, 42].

The use of a viral vector to deliver a known IOP lowering transgene to the anterior chamber has several advantages over existing therapeutic approaches to reduce IOP in a sustained fashion. Just as the first-line treatment for GON or ocular hypertension in many practices is the daily topical application of PGF2α analogues including latanoprost and bimatoprost [38], at present, the leading two candidates for sustained IOP reduction are both based on elution of bimatoprost from a scaffold. The Bimatoprost Ocular Ring (Allergan) is a polypropylene and silicone matrix ring containing bimatoprost that is placed into the conjunctival fornix by a physician. Drug is released into the tear film in a descending dose manner given the passive, concentration gradient driven diffusion of drug [61]. Since the drug is eluted into the tear film, ocular surface side effects may still be observed. Other reported issues with this device include insert-related ocular or peri-ocular discomfort and dislodgment of the device [61]. The other leading candidate for sustained IOP reduction is the recent FDA approved bimatoprost sustained-release implant (Bimatoprost SR, Durysta, Allergan). The implant is composed of a polymer matrix containing bimatoprost that is delivered by an injector into the anterior chamber where it slowly degrades releasing the drug [62, 63]. Up to half of patients receiving Bimatoprost SR have experienced some form of ocular surface side effect including conjunctival hyperemia, eye irritation or eye pain [64]. Perhaps the greatest concern of this approach is the free-floating nature of this device in the anterior chamber. In a 20-month study following patients with Bimatoprost SR, over 10% of patients had a greater than 20% loss of corneal endothelial cells [64]. In the case of transgene expression of STC-1-FLAG, a different pharmacokinetic response is observed based on continued expression of the transgene. Furthermore, being a secreted-protein, no drug eluting scaffold or device is required for a treatment based on STC-1-FLAG transgene expression.

In summary, transgene expression of the peptide hormone STC-1 represents a novel approach to sustained IOP reduction therapy that may provide a safe and effective treatment strategy for the 80 million worldwide affected by glaucoma that does not require the use of the FP receptor or a drug elution scaffold for IOP reduction.

## Supporting information

S1 Fig Transgene expression of STC-1-FLAG by PCR. ssAAV2-STC-1-FLAG was injected in one eye and PBS was injected into the fellow eye of 3-month-old C57BL/6J mice (n = 12, 1 μL, intracameral injection, 3x10^9^ VGs). At the end of experimental week 28, all remaining mice in the study received a second injection. At experimental week 38, tissues were collected. In eyes receiving ssAAV2-STC-1-FLAG injections, PCR amplification produced a 728 base pair band consistent with STC-1-FLAG transcripts.
(TIF)

S2 Fig STC-1-FLAG expression at 18 weeks. Nine weeks after injection with ssAAV2-STC-1-FLAG (3x10^9^ VGs) in one eye and ssAAV2-GFP (3x10^9^ VGs) in the fellow eye, remaining mice in the study (n = 8) were randomized to continue without further treatment (n = 4) or receive a second injection. At week 18, STC-1-FLAG iridocorneal angle including ciliary body (CB); the anterior segment including cornea (C), iris (I), and lens capsule (L); and retina. Mice that had received a second injection of ssAAV2-STC-1-FLAG showed enhanced staining for STC-1-FLAG suggesting increased expression. Note mild autofluorescence in STC-1-FLAG controls.
(TIF)

S3 Fig ssAAV2-STC-1-FLAG does not affect episcleral venous pressure or uveoscleral outflow. 3-month-old C57BL/6J mice were randomized to receive either a single injection of intracameral ssAAV2-STC-1-FLAG (n = 5;3x10^9^ VG)) or ssAAV2-GFP (n = 5; (3x10^9^ VGs) or daily topical STC-1-FLAG (n = 4), LFA (n = 5) or no treatment (n = 4) in one eye. Aqueous humor dynamics was performed at maximal treatment response (week 6 for injection experiments and day 5 for topical experiments). A) No significant difference among any treatment groups was seen with respect to episcleral venous pressure, B) uveoscleral outflow, or C) Aqueous humor formation rate.
(TIF)

S1 Dataset (PDF)

S1 Raw image (PDF)
