# Supplementary figures and images for: Transgene expression of Stanniocalcin-1 provides sustained intraocular pressure reduction by increasing outflow facility
Source: PLoS One. 2022 May 31;17(5):e0269261. doi: 10.1371/journal.pone.0269261 (PMC9154118; doi:10.1371/journal.pone.0269261)

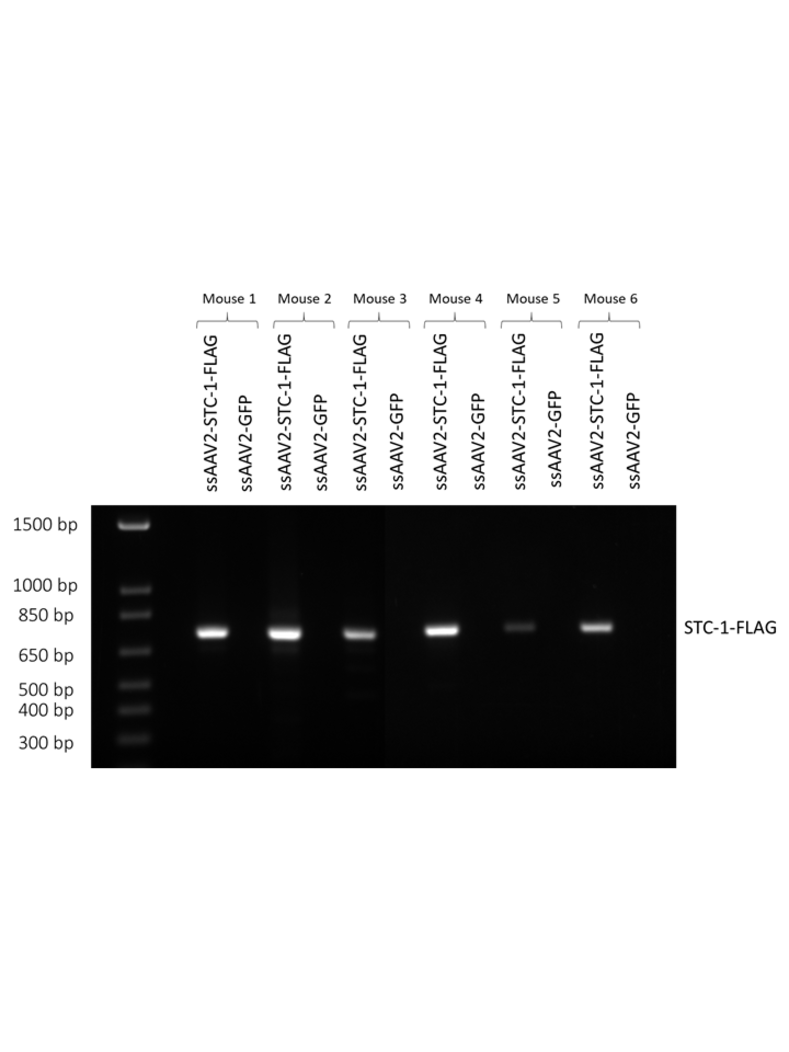

Supplement: S1 Fig — ssAAV2-STC-1-FLAG was injected in one eye and PBS was injected into the fellow eye of 3-month-old C57BL/6J mice (n = 12, 1 μL, intracameral injection, 3x10^9^ VGs). At the end of experimental week 28, all remaining mice in the study received a second injection. At experimental week 38, tissues were collected. In eyes receiving ssAAV2-STC-1-FLAG injections, PCR amplification produced a 728 base pair band consistent with STC-1-FLAG transcripts. (TIF) [file pone.0269261.s001.tif]

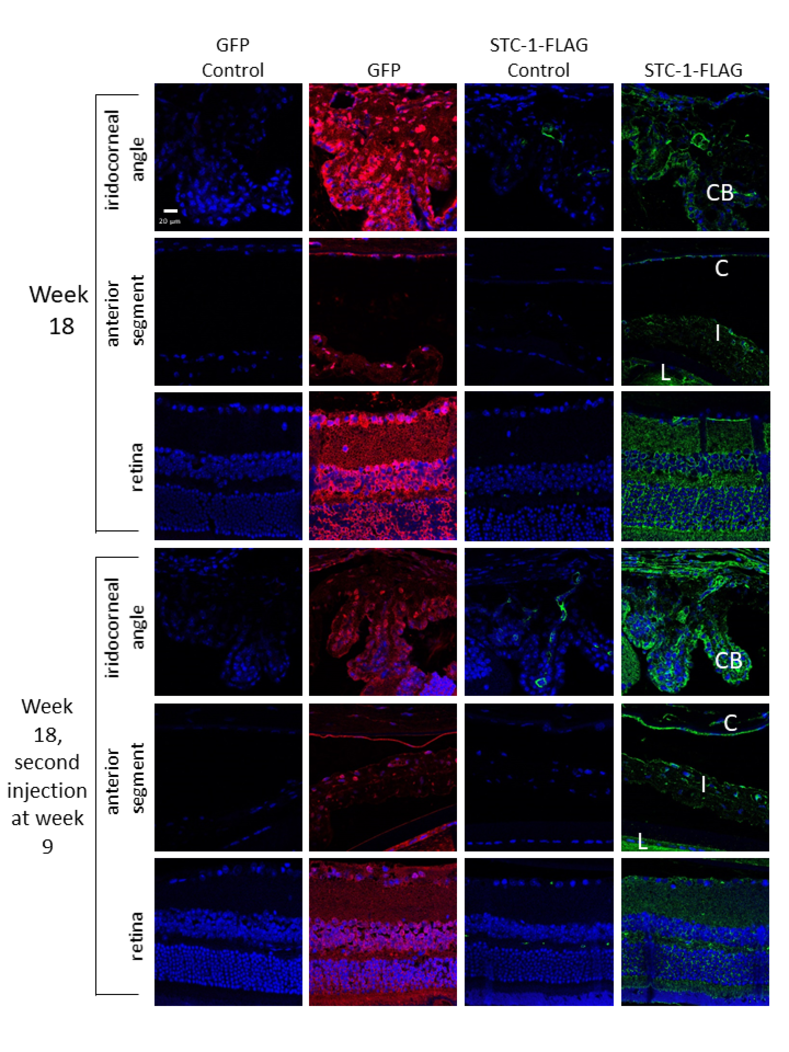

Supplement: S2 Fig — Nine weeks after injection with ssAAV2-STC-1-FLAG (3x10^9^ VGs) in one eye and ssAAV2-GFP (3x10^9^ VGs) in the fellow eye, remaining mice in the study (n = 8) were randomized to continue without further treatment (n = 4) or receive a second injection. At week 18, STC-1-FLAG iridocorneal angle including ciliary body (CB); the anterior segment including cornea (C), iris (I), and lens capsule (L); and retina. Mice that had received a second injection of ssAAV2-STC-1-FLAG showed enhanced staining for STC-1-FLAG suggesting increased expression. Note mild autofluorescence in STC-1-FLAG controls. (TIF) [file pone.0269261.s002.tif]

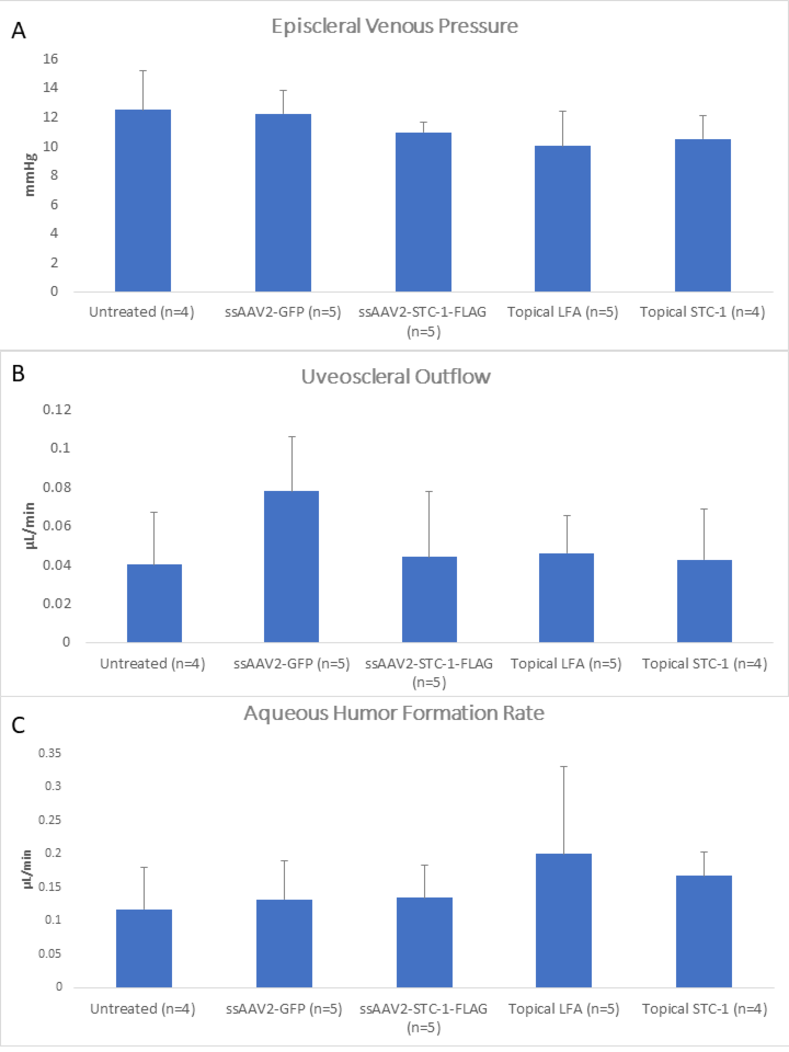

Supplement: S3 Fig — 3-month-old C57BL/6J mice were randomized to receive either a single injection of intracameral ssAAV2-STC-1-FLAG (n = 5;3x10^9^ VG)) or ssAAV2-GFP (n = 5; (3x10^9^ VGs) or daily topical STC-1-FLAG (n = 4), LFA (n = 5) or no treatment (n = 4) in one eye. Aqueous humor dynamics was performed at maximal treatment response (week 6 for injection experiments and day 5 for topical experiments). A) No significant difference among any treatment groups was seen with respect to episcleral venous pressure, B) uveoscleral outflow, or C) Aqueous humor formation rate. (TIF) [file pone.0269261.s003.tif]

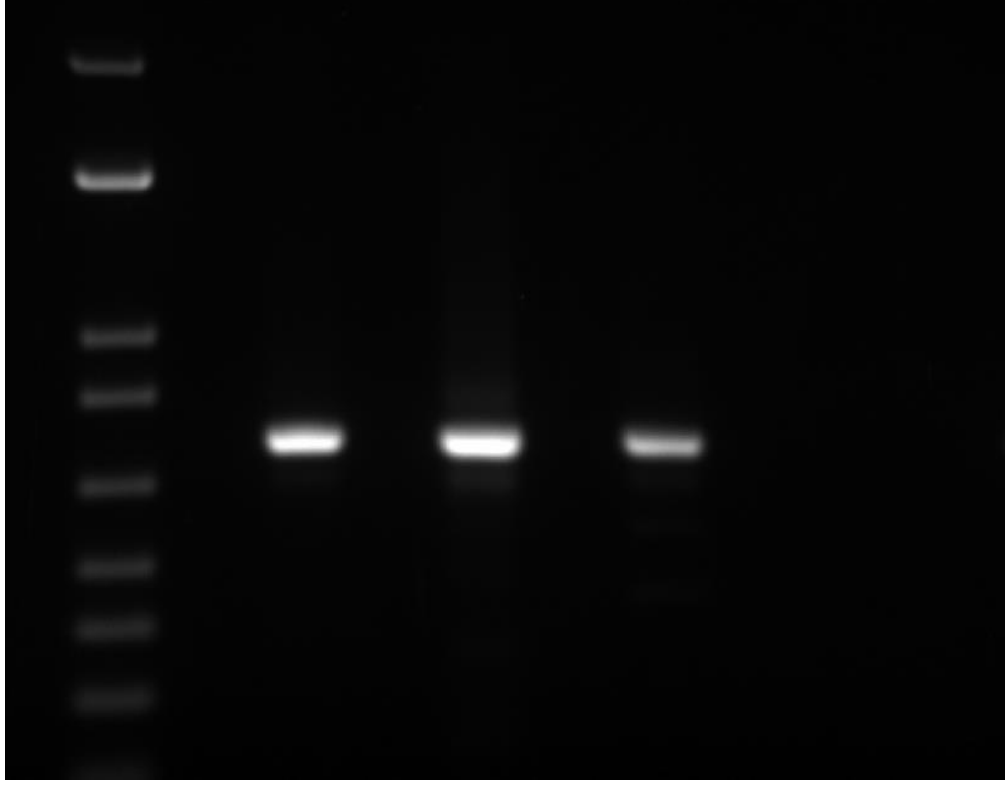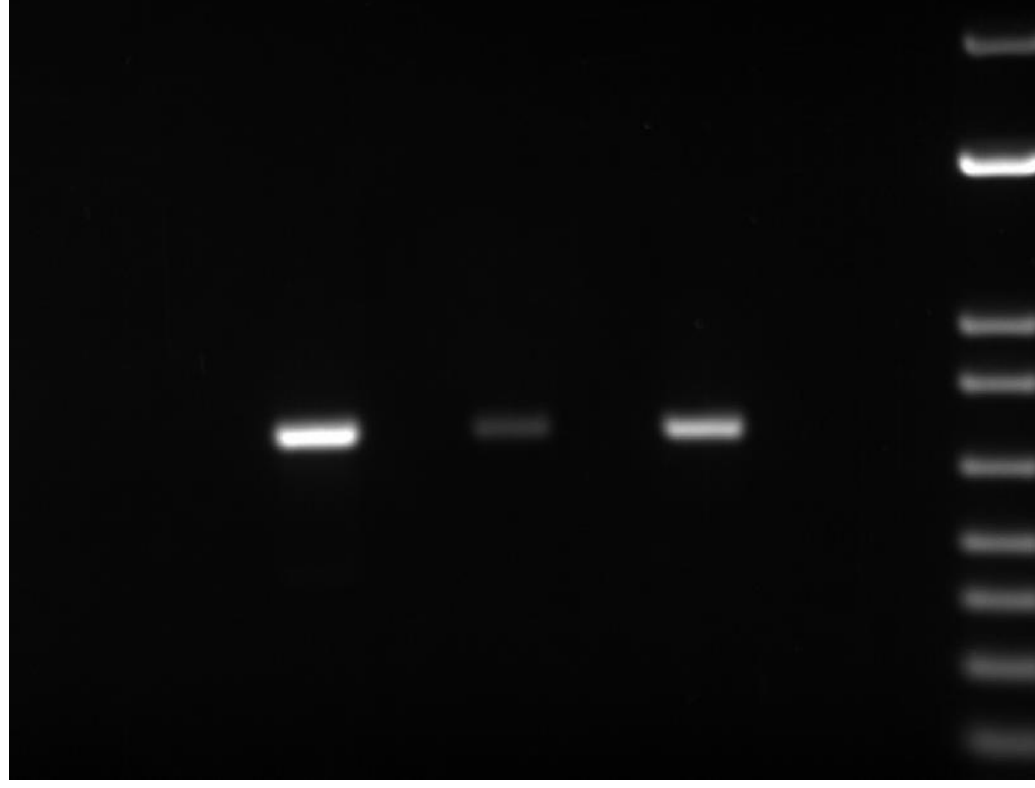

Supplement: S1 Raw image — (PDF) [file pone.0269261.s005.pdf]
