# Supplementary material for: Transgene expression of Stanniocalcin-1 provides sustained intraocular pressure reduction by increasing outflow facility
Source: PLoS One. 2022 May 31;17(5):e0269261. doi: 10.1371/journal.pone.0269261 (PMC9154118; doi:10.1371/journal.pone.0269261)
Supplement: S1 Dataset — (PDF) [file pone.0269261.s004.pdf]

GW Roddy et al. *Transgene expression of Stanniocalcin-1 provides sustained intraocular pressure reduction by increasing outflow facility*

Exp 1 (Fig 1)

	Baseline	Days post-injection			
Days	n=26		n=16	n=8	
	Baseline	1	2	3	4
ssAAV2-GFP inj	16.60	13.97	16.60	16.75	17.35
ssAAV2-STC-1-F	16.55	13.47	13.36	14.67	14.58
% Change	-0.31	-3.62	-19.51	-12.44	-15.97
P-Value	0.43	0.54	0.00	0.00	0.00

Exp 2 (Fig 3)

		Month 1									
Average IOP in	Baseline	1.0	2.0	3.0	4.0	5.0	6.0	7.0	7.5-8*	8*	9*
PBS Injected	16.41	16.82	16.23	16.95	16.34	16.71	16.40	16.09	16.18	16.52	16.90
ssAAV2-STC-1-F	16.56	14.10	13.56	14.45	14.25	15.07	15.01	15.06	12.79	13.06	13.33
% change	1.01	-16.19	-16.30	-14.70	-13.10	-9.66	-8.37	-6.22	-20.85	-20.85	-21.21
P-Value	0.21	0.00	0.00	0.00	0.00	0.00	0.00	0.01	0.00	0.00	0.00

Exp 3 (Fig 4)

		n=12							n=8		Re-Injected (n=4)				
	Baseline	1	2	3	4	5	6	7	8	9	10	12	14	16	18
ssAAV2-GFP inj	16.78	16.44	16.90	17.06	16.74	16.35	15.93	16.24	15.96	16.52	16.92	16.00	16.92	16.96	16.38
ssAAV2-STC-1-F	16.74	14.50	13.97	14.03	14.57	13.82	14.24	13.72	13.71	14.15	14.33	13.58	14.00	13.13	13.44
Re-Injected ssAAV2-GFP (n=4)										16.52	16.29	16.79	16.46	16.56	16.73
Re-Injected ssAAV2-STC-1-FLAG (n=4)										14.15	11.25	12.04	13.17	14.04	13.52

Exp 4 (Fig 6)

Average IOP in	Baseline	1	4	8	14
ssAAV2-GFP	16.28	16.32	17.15	16.22	16.63
ssAAV2-STC-1-F	16.34	13.98	14.22	13.35	14.95
% change	0.57	-14.10	-16.98	-17.67	-10.05
P-Value	0.82	0.01	0.00	0.00	0.00

Experiment 5 (Fig 7, S3)

Outflow facility

	AV	Std Dev
Untreated (n=4	0.025	0.006
ssAAV2-GFP (n=	0.016	0.005
ssAAV2-STC-1-F	0.038	0.008
Topical LFA (n=	0.046	0.018
Topical STC-1 (r	0.048	0.015

Uveoscleral

	AV	Std Dev
Untreated (n=4	0.040	0.027
ssAAV2-GFP (n=	0.078	0.028
ssAAV2-STC-1-F	0.044	0.034

Topical LFA (n=	0.046	0.019
Topical STC-1 (r	0.043	0.026

EVP

	AV	Std Dev
Untreated (n=4	12.505	2.689
ssAAV2-GFP (n=	12.212	1.611
ssAAV2-STC-1-I	10.960	0.657
Topical LFA (n=	10.002	2.362
Topical STC-1 (r	10.483	1.632

Aqueous formation

	AV	Std Dev
Untreated (n=4	0.118	0.063
ssAAV2-GFP (n=	0.132	0.058
ssAAV2-STC-1-I	0.134	0.050
Topical LFA (n=	0.200	0.131
Topical STC-1 (r	0.168	0.036

Measured IOP

	AV	Std Dev
Untreated (n=4	15.915	0.499
ssAAV2-GFP (n=	16.000	1.225
ssAAV2-STC-1-I	13.400	0.548
Topical LFA (n=	13.200	0.899
Topical STC-1 (r	13.230	0.632
